# Thermal Annealing Effect on Poly(3-hexylthiophene): Fullerene:Copper-Phthalocyanine Ternary Photoactive Layer

**DOI:** 10.1155/2013/914981

**Published:** 2013-05-20

**Authors:** H. Derouiche, A. B. Mohamed

**Affiliations:** Laboratoire de Photovoltaïques, Centre de Recherches et des Technologies de l'Energie, BP 95, 2050 Hammam-Lif, Tunisia

## Abstract

We have fabricated poly(3-hexylthiophene) (P3HT)/copper phthalocyanine (CuPc)/fullerene (C60) ternary blend films. This photoactive layer is sandwiched between an indium tin oxide (ITO)/poly(3,4-ethylenedioxythiophene):poly(styrene sulfonate) (PEDOT/PSS) photoanode and a bathocuproine (BCP)/aluminium photocathode. The thin films have been characterized by atomic force microscope (AFM) and ultraviolet/visible spectroscopy in order to study the influence of P3HT doping on the morphological and optical properties of the photoactive layer. We have also compared the *I*-*V* characteristics of three different organic solar cells: ITO/PEDOT:PSS/CuPc_0.5_:C60_0.5_/BCP/Al and ITO/PEDOT:PSS/P3HT_0.3_:CuPc_0.3_:C60_0.4_/BCP/Al with and without annealing. Both structures show good photovoltaic behaviour. Indeed, the incorporation of P3HT into CuPc:C60 thin film improves all the photovoltaic characteristics. We have also seen that thermal annealing significantly improves the optical absorption ability and stabilizes the organic solar cells making it more robust to chemical degradation.

## 1. Introduction

Thin films composed of copper phthalocyanine CuPc and fullerene C60, p-n small molecules, have in recent years received increasing attention, due to new optoelectronic applications [1–4]. CuPc:C60 composite film was commonly used to obtain a high conversion efficiency of solar energy into electrical energy [5–8]. However, there is incomplete knowledge about physical and chemical mechanisms of organic thin films stability [9–11].

Addition of polymer is proposed as a way to stabilize the CuPc:C60 thin films, making it more robust to chemical degradation. Regioregular poly(3-hexylthiophene) (P3HT) polymers are widely used in organic devices [12–15]. P3HT has a good chemical stability and good solubility in various solvents, so the addition of P3HT to the CuPc:C60 thin films improves the p-n bulk heterojunction.

Moreover, thermal annealing is an effective method that improves the morphology, compactness, and crystallinity of the organic thin films [16–19]. One of the most important effects of thermal annealing is the reorientation of the CuPc planar and C60 near-spherical shape small molecules in a high density P3HT matrix.

The aim of the present work is to study the influence of P3HT doping and thermal annealing on the structural, optical, and stability of the fullerene-based solar cells.

## 2. Experimental Details

**Figure 1:**
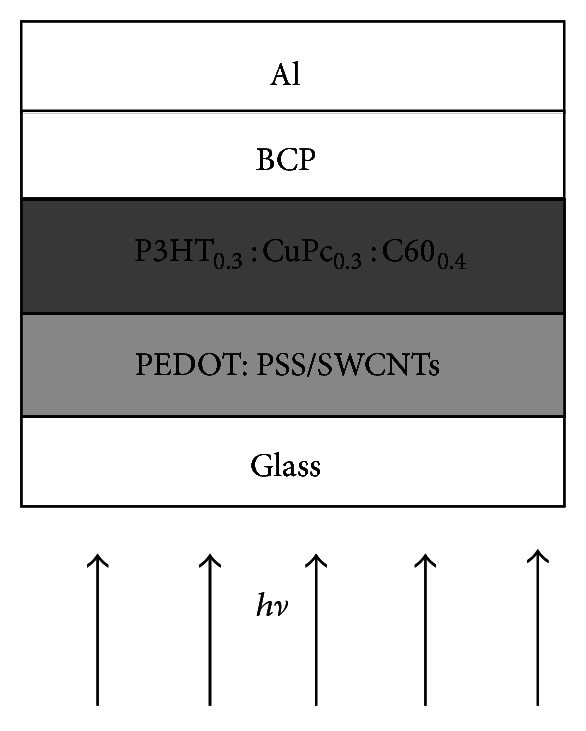
Structure of ITO/PEDOT:PSS/C60:CuPc:P3HT/BCP/Al solar cell.

All the organic layers have been deposited in a sandwich geometry between the two electrodes: indium tin oxide (ITO) as photoanode and aluminum as photocathode (Figure 1).

The ITO-coated glass substrates (Aldrich, surface resistivity 8–12 *Ω*/sq) were treated with an 80°C H_2_O-H_2_O_2_ (30%)-NH_4_OH (25%) solution (5 : 1 : 1 vol. parts) for 20 min and then cleaned in sequence with acetone (15 min) and isopropanol (15 min) and deionized water (15 min) using an ultrasonic bath.

PEDOT:PSS, 0.54% in H_2_O, high-conductivity grade with 1–5% Propan-2-ol, and 5–10% diethylene glycol were purchased from Aldrich. It is the most hole transparent conductor used as buffer layer, having a good charge transport properties (resistance of 200 *Ω*/sq). In addition, single-walled carbon nanotubes (SWNTs) were dispersed in a PEDOT:PSS solution with a weight ratio of 0.5 wt%, and the obtained solutions were spin coated onto ITO.

As for CuPc, C60, and P3HT, they were purchased from Aldrich and used without further purification. In this work, polymer solutions were prepared by dissolving P3HT/CuPc/C60 with weight ratios of 30 : 30 : 40 in dichlorobenzene. The thickness was checked by cross- section visualization using SEM.

In addition, bathocuproine (BCP) and aluminum upper contacts were deposited onto the organic layers by vacuum evaporation at a pressure of 10^−4^ Pa. A mask was used to determine a well-defined shape for the aluminum electrode.

Finally, to increase the stability of the organic cell, polyvinylidene difluoride (PVDF) film was put on top of the organic solar cell and annealed for 5 min at 70°C. This encapsulation method limits diffusion of oxygen and water into the active layer.

## 3. Results and Discussion

**Figure 2:**
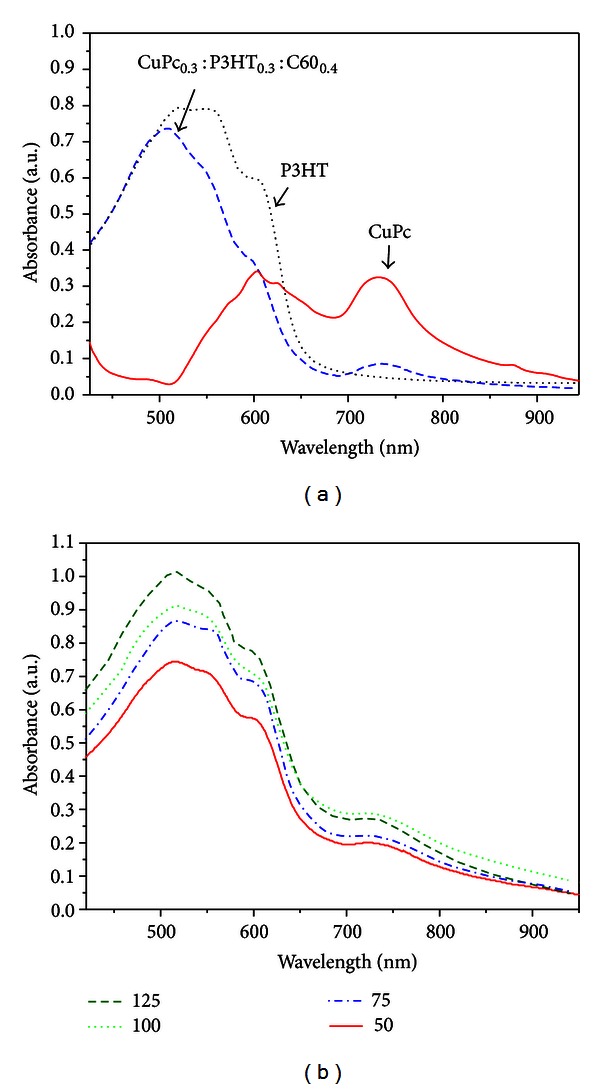
The absorbance spectra of (a) CuPc (solid line), P3HT (dotted line), and P3HT_0.3_:CuPc_0.3_:C60_0.4_ before annealing (dashed line) and (b) P3HT_0.3_:CuPc_0.3_:C60_0.4_ after annealing at different temperatures.

Figure 2(a) shows the absorption spectra of a CuPc, P3HT, and P3HT_0.3_:CuPc_0.3_:C60_0.4_ films. CuPc has a strong absorption in the visible region, with the Q-band absorption peaks at around 620 and 740 nm related to the *π*-π* transition [20]. For pure P3HT film, the absorption spectra showed two peaks at 493 nm and 517 nm and one shoulder at 572 nm. These three bands can be attributed to the *π*-π* transition [21]. As for P3HT_0.3_:CuPc_0.3_:C60_0.4_ ternary blend bulk heterojunction, broadening and splitting of the absorption spectrum are observed. The presence of the strong vibronic feature near 600 nm is attributed to P3HT interchain interaction [22].

Figure 2(b) shows the effect of thermal annealing on the UV-Vis absorption spectra for the thin films of P3HT_0.3_:CuPc_0.3_:C60_0.4_. The annealing time here is 15 min for all the films. Optical intensities of the peaks at both 550 nm and 600 nm correspondent to P3HT were improved as the annealing temperature is increased; this indicates an enhanced ordering of the organic materials in the thin film [23]. However, the optical intensity of the peaks at 740 nm correspondent to CuPc decreased as the annealing temperatures were higher than 100°C. The degradation is possibly due to the change in orientation of the CuPc planar and C60 nearspherical shape small molecules in the P3HT_0.3_:CuPc_0.3_:C60_0.4_ thin films.

**Figure 3:**
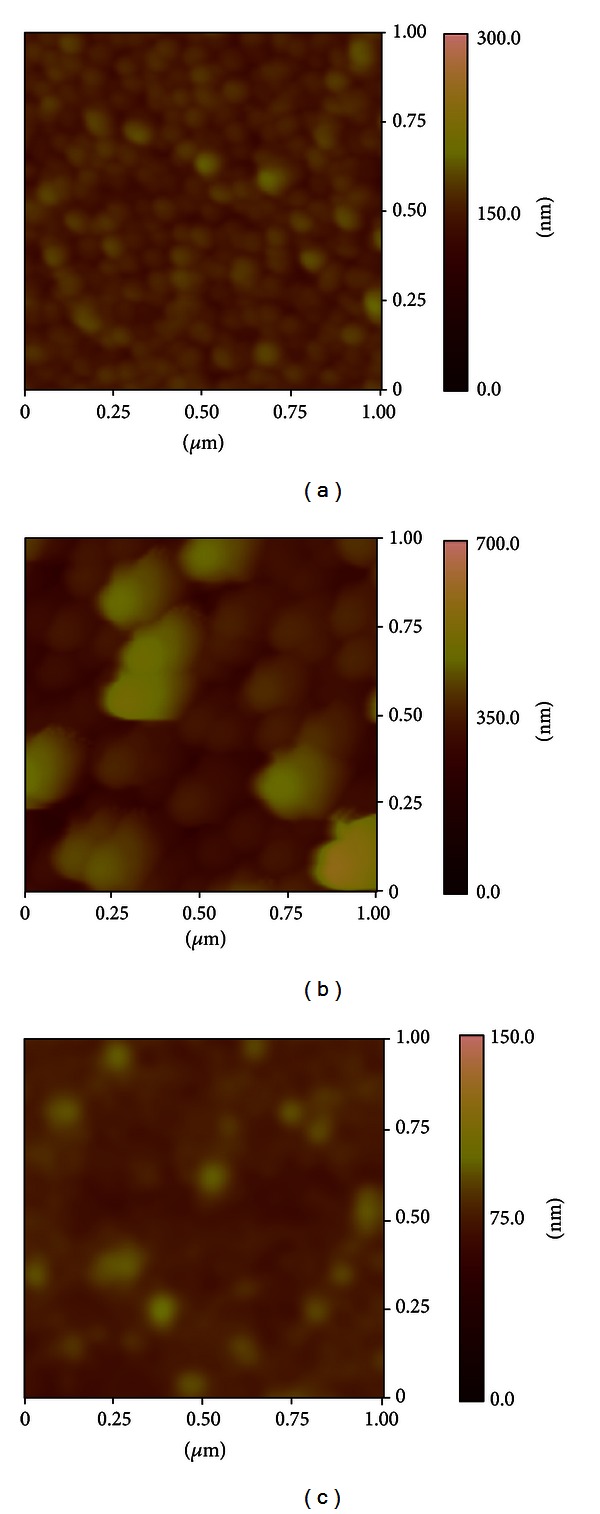
AFM image of (a) C60, (b) CuPc:C60, and (c) P3HT_0.3_:CuPc_0.3_:C60_0.4_ thin films.

AFM (Figure 3) observations allow visualizing of the surface structure of C60, CuPc_0.5_:C60_0.5_, and P3HT_0.3_:CuPc_0.3_:C60_0.4_ thin films after thermal annealing for 15 min at 100°C. Fullerene C60 thin films (Figure 3(a)) form crystalline structures. After the addition of CuPc (Figure 3(b)), CuPc_0.5_:C60_0.5_ clearly show the presence of dense packing film with a smooth surface and demonstrate highly ordered surface composed of C60 agglomeration and CuPc molecular planes. AFM images of C60, CuPc_0.5_:C60_0.5_ (Figure 3(c)) show that the formation of an amorphous thin film composed of C60:CuPc agglomerates into an amorphous matrix of P3HT.

**Figure 4:**
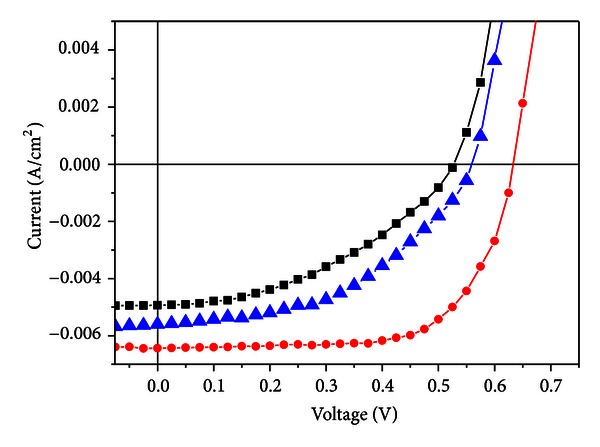
*I*  (*V*) characteristics of (i) ITO/PEDOT:PSS/P3HT_0.3_:CuPc_0.3_:C60_0.4_/BCP/Al heat-treated (●), (ii) ITO/PEDOT:PSS/P3HT_0.3_:CuPc_0.3_:C60_0.4_/BCP/Al (▲), and (iii) ITO/PEDOT:PSS/CuPc_0.5_:C60_0.5_/BCP/Al (■).

Typical *I*-*V* measurements under AM 1.5 illuminations of the devices are plotted in Figure 4. ITO/PEDOT:PSS/CuPc_0.5_:C60_0.5_/BCP/Al, we have a *V*_OC_ of 0.52 V, an *I*_SC_ of 4.93 mA/cm², and a fill factor of 0.4 giving energy conversion efficiency of 1.02%. For the ITO/PEDOT:PSS/P3HT_0.3_:CuPc_0.3_:C60_0.4_/BCP/Al device develops an open circuit voltage *V*_OC_ of 0.55 V, an *I*_SC_ of 5.7 mA/cm², and a fill factor of 0.46 giving energy conversion efficiency of 1.44%.

All structures show significant photovoltaic behaviour. The most remarkable feature is an increase in the short circuit current at P3HT doping. In fact, under illumination, a fraction of incorporated P3HT polymers improve photon absorption of the CuPc system leading to a higher number of free holes in the valence band, which establishes a p-type doping inside the materials. Furthermore, P3HT polymers can also act as exciton dissociation sites and therefore generate free charge carriers in the presence of excitons. On the other hand, with P3HT, both the open-circuit voltage and the fill factor increase, respectively, to a value around 0.55 V and 0.46 compared to 0.52 V and 0.4. Therefore, we can note that the open-circuit voltage and the fill factor depend on the nature of the photoactive polymers.

After thermal annealing of the photoactive layer for 15 min at 100°C, ITO/PEDOT:PSS/P3HT_0.3_:CuPc_0.3_:C60_0.4_/BCP/Al device develops an open circuit voltage *V*_OC_ of 0.63 V, a short circuit current *I*_SC_ of 6.5 mA/cm², a fill factor of 0.68, and an energy conversion efficiency *η* = 2.8%. As a result of heat treatment, the organic solar cell performance was dramatically improved. We can conclude that thermal annealing enhances photovoltaic characteristics by optimizing both the morphology and the stability of the photoactive layer.

**Figure 5:**
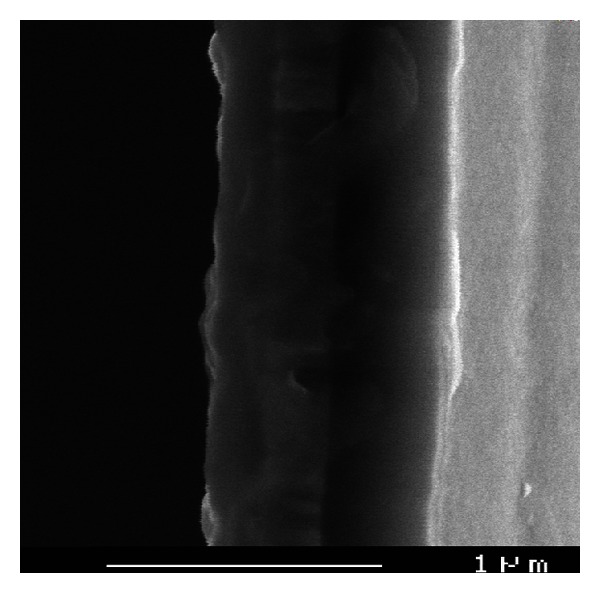
Cross-sectional SEM micrograph of glass/ITO/PEDOT: PSS/P3HT_0.3_:CuPc_0.3_:C60_0.4_.

Finely, cross-sectional SEM micrograph of glass/ITO/PEDOT:PSS/P3HT_0.3_:CuPc_0.3_:C60_0.4_ structure was checked by cross-section visualization using a scanning electron microscope (SEM) after one month under both environmental and light exposure (Figure 5). We can see that there are no chemical degradation and phase segregation. We can note that the addition of P3HT stabilizes the photoactive layer by reducing the density of structural defects and phase segregation.

## 4. Conclusions

The addition of P3HT in CuPc:C60 thin films has been studied by *I*-*V* measurements under AM 1.5 illumination. The incorporation of P3HT in the films improved the optical properties of the CuPc-C60 thin films and increased the solar cell parameters values (*I*_SC_ and *η*) from 4.93 to 5.7 mA/cm² and from 1.02 to 1.44%, respectively. In addition, thermal annealing, for 15 min at 100°C, stabilizes the bulk heterojunction photoactive layer making it more robust to chemical degradation under prolonged operation and improves the power conversion efficiency to 2.8%.
